# Perpendicular and turbulent flow after aortic valve replacement: paravalvular or transvalvular leakage? – a case report

**DOI:** 10.1186/s13019-020-1050-4

**Published:** 2020-01-14

**Authors:** Shihoko Iwata, Chiaki Inano, Makoto Ozaki

**Affiliations:** Department of Anesthesiology, Tokyo Women’s Medical University Hospital, 8-1 Kawada-cho, Shinjuku-ku, Tokyo, 162-8666 Japan

**Keywords:** Paravalvular leakage, Transvalvular leakage, Intraoperative transesophageal echocardiography, Biological valve, Extra cardiopulmonary bypass, Aortic valve replacement

## Abstract

**Background:**

Perpendicular transvalvular leakage (TVL) is occasionally observed after aortic valve replacement (AVR) in biological valves with a stent post, often originating from the base of the stent post. However, an observed perpendicular jet flow is not always a TVL. In rare cases, paravalvular leakages (PVLs) can be perpendicular and are present behind a TVL. In the present case, both PVL and TVL existed simultaneously as unusual perpendicular jet flows that originated from sites in close proximity to the stent post.

**Case presentation:**

A 73-year-old man underwent AVR with a biological valve in the supra-annular position using the non-everting mattress suture technique with pledgets. After weaning from cardiopulmonary bypass (CPB), transesophageal echocardiography (TEE) revealed an unfamiliar perpendicular turbulent flow, similar to reported TVL, originating from the anterior stent post. Further TEE examination revealed a PVL had originated from the site between the sewing ring at the anterior stent post and native annulus attached to a pledget. The space between the sewing ring and annular retained native portion caused the perpendicular turbulent jet. Consequently, two types of perpendicular turbulent flows, TVL and PVL, existed adjacent to each other.

After reinstitution of CPB, inspection of the prosthesis itself indicated it to be normal, but there was a region adjacent to the anterior stent post near the right coronary ostium where the tip of the curved Pean forceps entered between the sewing ring and the native annulus. The region was consistent with TEE findings. AVR was performed with the same prosthesis again. After weaning from CPB, immediate TEE revealed that the unusual perpendicular turbulent flows had disappeared and only a few small TVLs were observed. Regarding the disappearance of TVL, we considered that the fabric region of the prosthetic valve was covered with cellular elements to prevent the leak, as it was already used in AVR once and soaked in blood.

**Conclusions:**

Perpendicular turbulent flow raises the possibility of both TVL and PVL in the case of AVR with stented bovine pericardial valves. For a differential diagnosis of TVL or PVL, it is important to know the surgical procedures and valve morphology.

## Background

Cardiovascular anesthesiologists occasionally find small leakages and backflow jets originating from the central coaptation point, the fabric-covered sites of the stent post, the region between the stent post and the sewing ring [1], or commissures between biological valves [2] after weaning from cardiopulmonary bypass (CPB). These jets are identified as transvalvular leakages (TVLs) that generally require follow-up monitoring. On the other hand, paravalvular leakage (PVL) is associated with morbidity [3, 4] and mortality [5], and requires immediately repair during extra CPB, especially if more-than-moderate regurgitation is detected after weaning from CPB. Therefore, it is important that PVL is immediately distinguished from TVL using intraoperative transesophageal echocardiography (TEE), even if both PVL and TVL are unfamiliar perpendicular jets and originate from the region near a stent post.

## Case presentation

A 73-year-old man (weight 73 kg, height 170 cm) with a history of hypertension was scheduled for aortic valve replacement (AVR) because of severe aortic regurgitation (AR). The patient was taken to the operating room and anesthetized with midazolam, remifentanil, and sevoflurane. After tracheal intubation, a TEE probe, X7-2t Live 3D TEE xMATRIX array transducer (Philips Ultrasound, Inc., Austin, TX) was inserted into the esophagus. An initial intraoperative TEE revealed severe AR. Spectral Doppler imaging revealed holodiastolic flow reversal in the descending thoracic aorta. In the midesophageal (ME) aortic valve (AV) long-axis (LAX), the aortic root diameter, sinus of Valsalva diameter, and diameter of the sinotubular junction were measured as being 3.0 cm, 4.0 cm, and 2.7 cm, respectively, and the vena contracta of eccentric AR was 5.3 mm with color flow Doppler (CFD). Moreover, TEE showed slightly reduced left ventricular motion (ejection fraction 39% in the modified Simpson method), trivial mitral regurgitation, and trivial tricuspid regurgitation. A patent foramen ovale (PFO) was confirmed using contrast TEE and CFD before CPB.

**Fig. 1:**
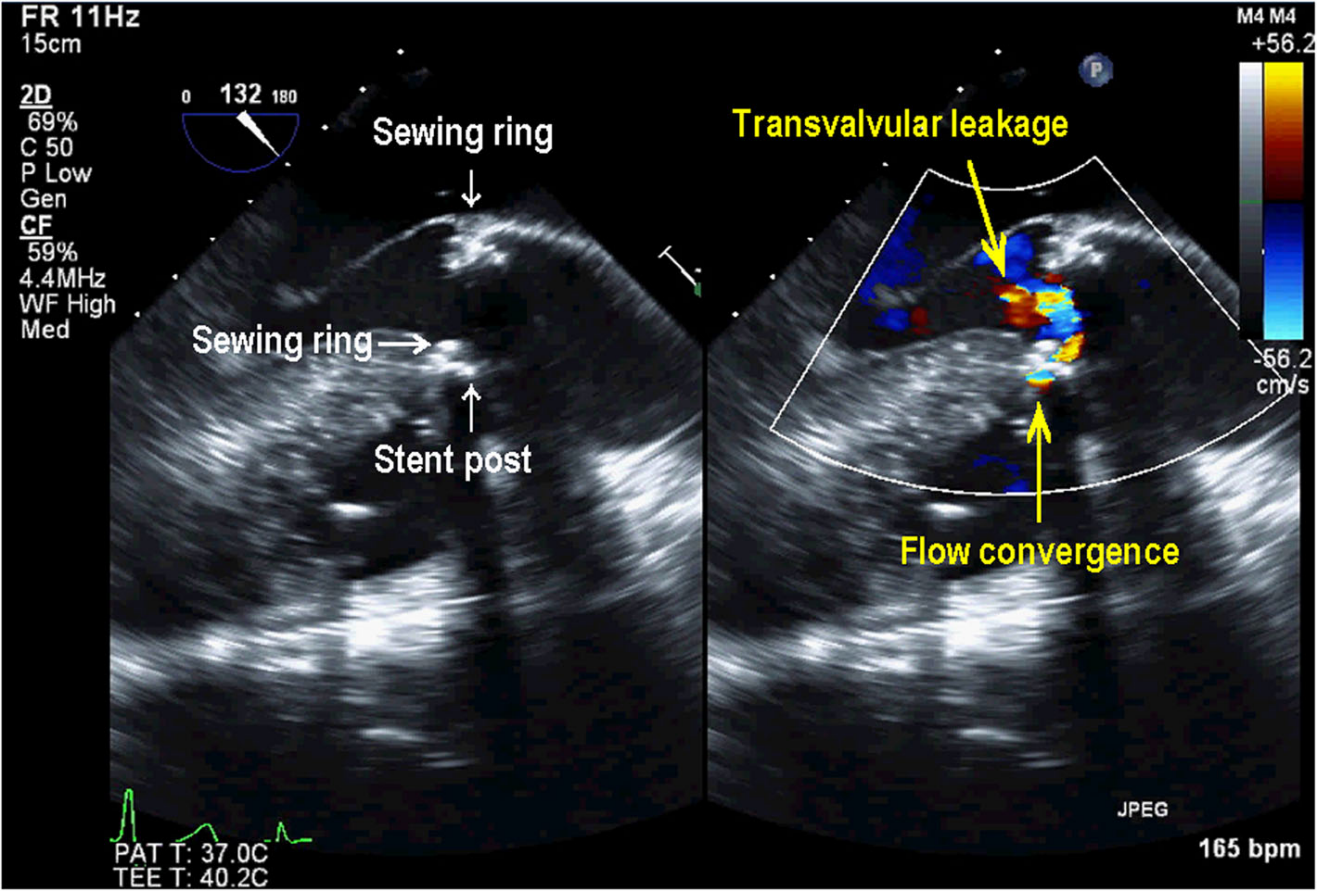
Midesophageal aortic valve long-axis image in color compare mode showing transvalvular leakage as the perpendicular turbulent jet that originated from between the stent post and sewing ring

**Fig. 2:**
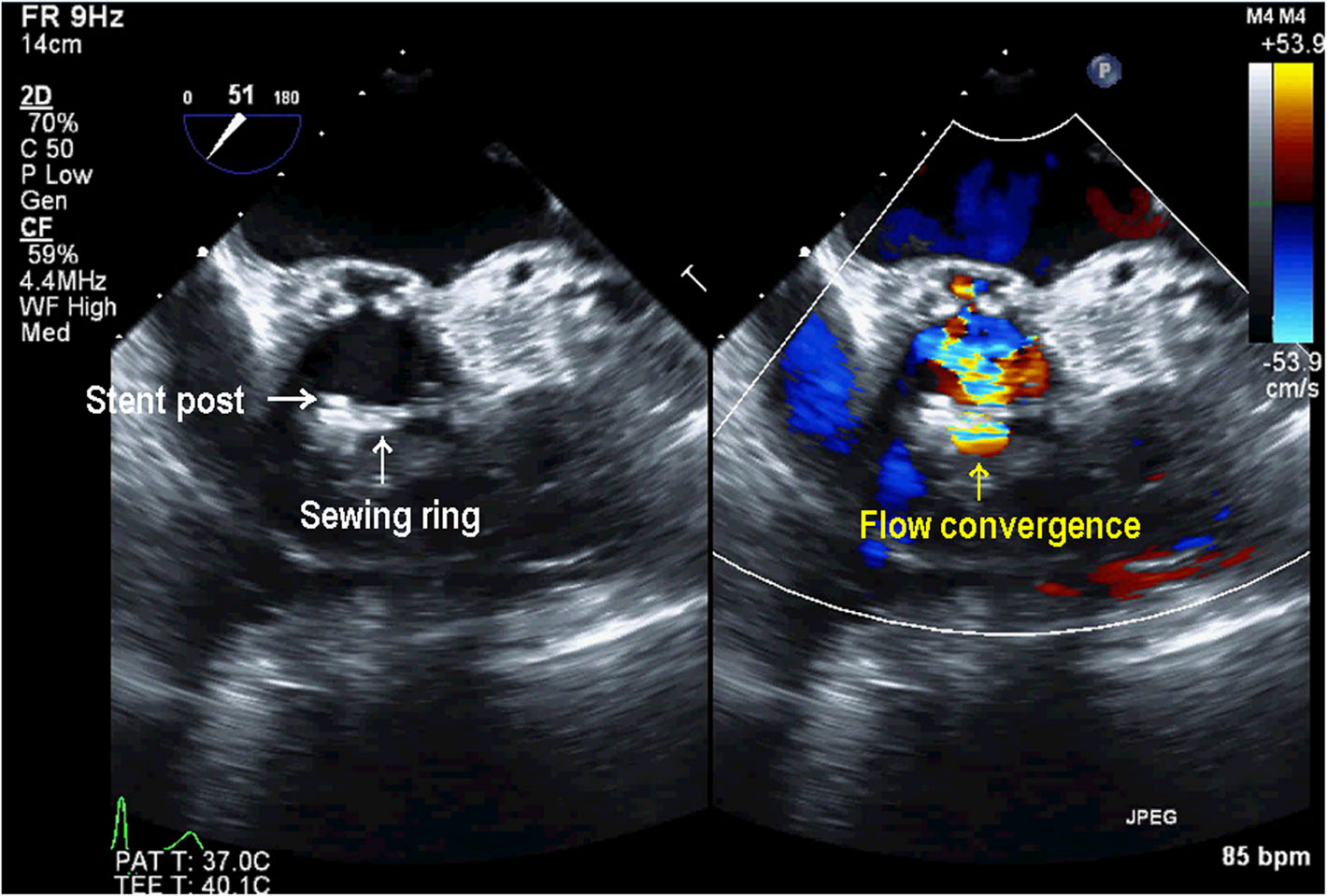
Midesophageal aortic valve short-axis image in color compare mode showing a considerable flow convergence adjacent to the stent post

**Fig. 3:**
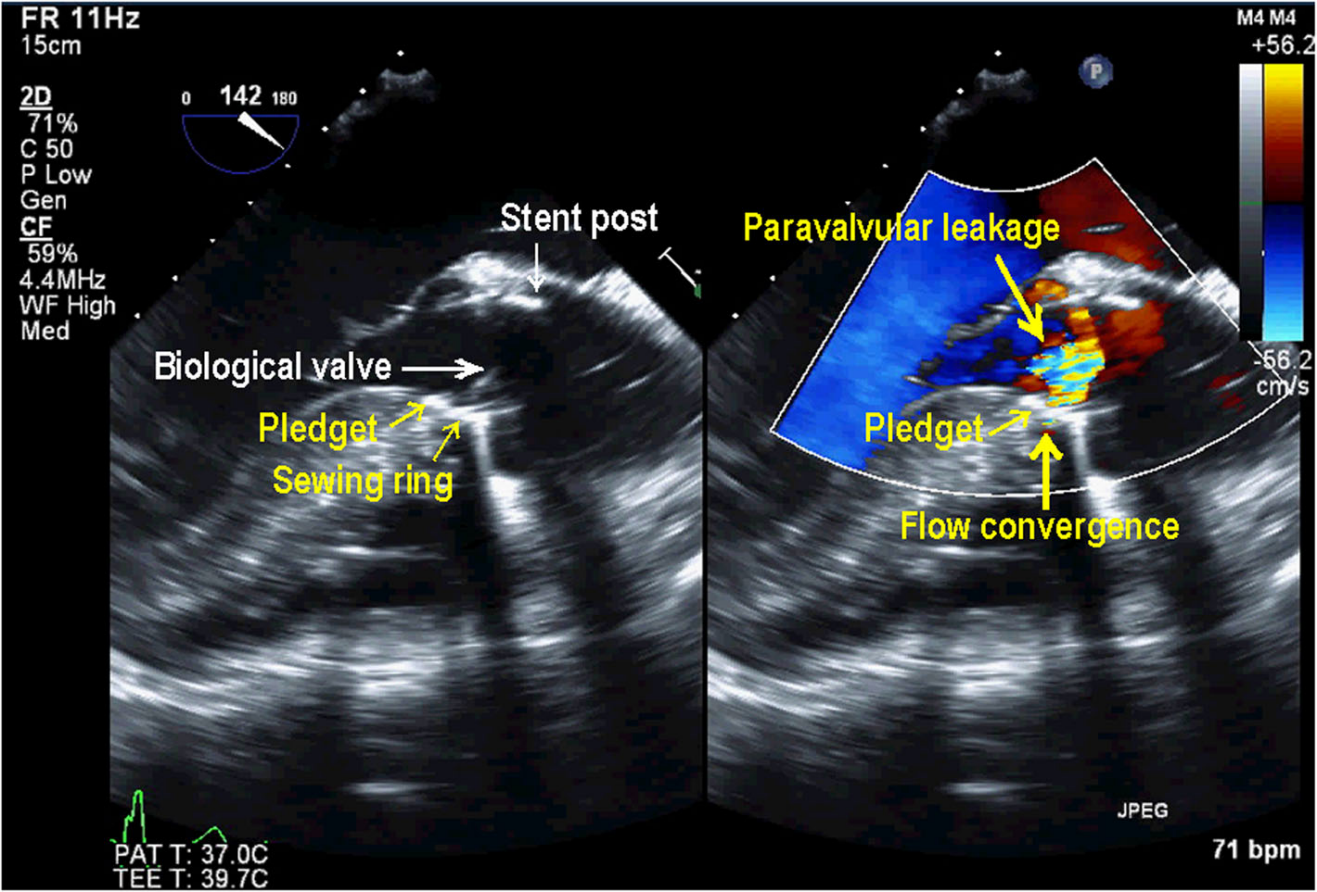
Midesophageal aortic valve long-axis image in color compare mode showing paravalvular leakage as turbulent flow from the outside of the sewing ring ran through perpendicularly below the sewing ring toward the left ventricular outflow tract on the opposite side

**Fig. 4:**
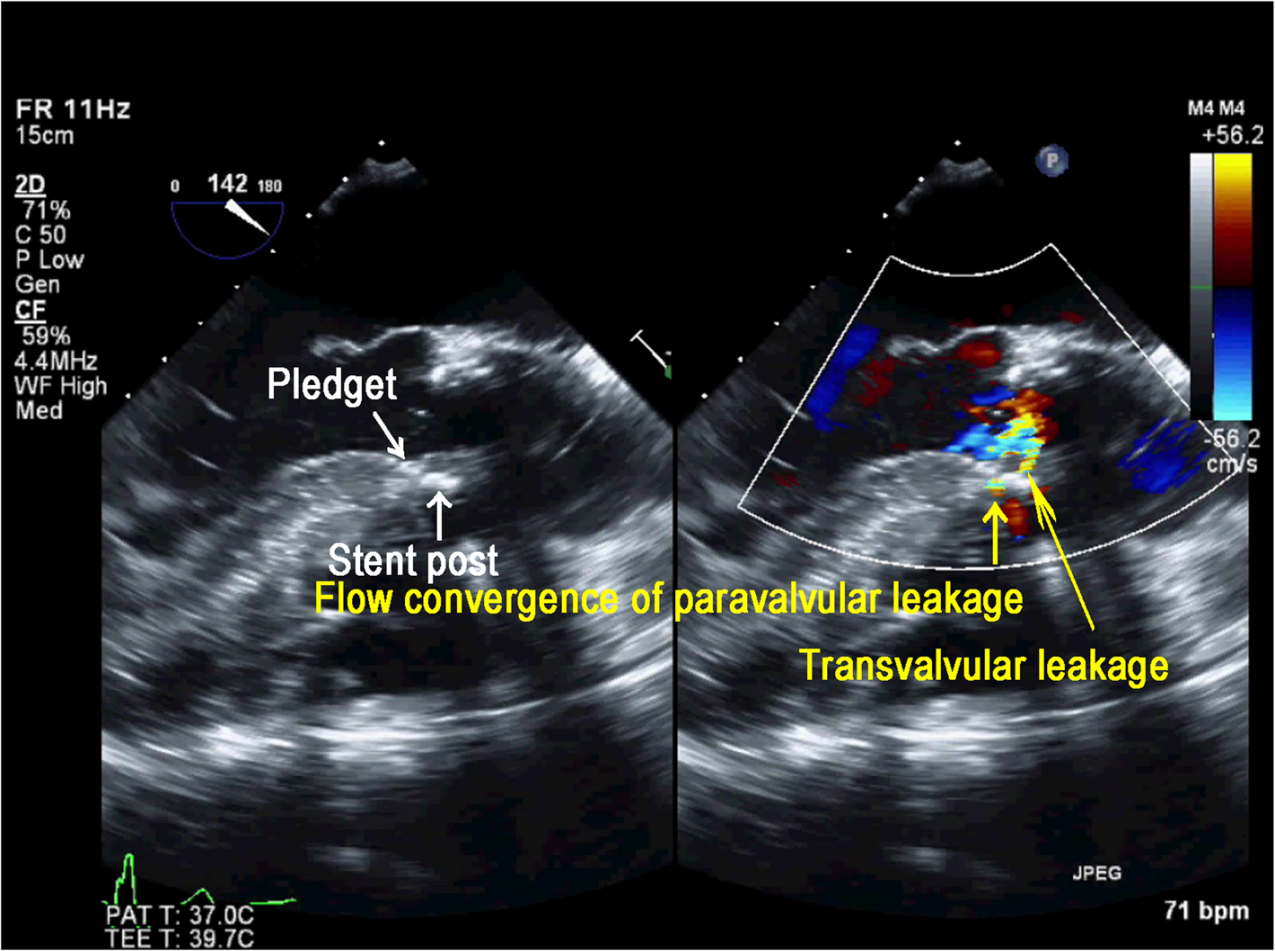
Midesophageal aortic valve long-axis image in color compare mode showing flow convergence of PVL originating from between a pledget and the sewing ring, and TVL from the stent post. TVL: transvalvular leakage, PVL: paravalvular leakage


AVR was performed in the supra-annular position with a 23-mm Carpentier-Edwards Perimount Magna Ease aortic valve (Edwards Lifesciences, Irvine, CA, USA) using the non-everting mattress suture technique with pledgets; the PFO was closed directly. The patient was weaned from CPB on low-dose dopamine and dobutamine. TEE confirmed a well-seated AV prosthesis with good mobility of the biological valve in the ME AV LAX and ME AV short-axis (SAX) views. However, CFD revealed perpendicular and turbulent flow from the sewing ring near the stent post to the opposite side in the ME LAX view (Fig. 1, Additional file 1: Video Clip S1). The color flow jet ran perpendicularly from above the sewing ring though between the stent post and the sewing ring, as characteristic of non-central non-PVLs [1]. However, in the ME AV SAX view, a considerable flow convergence was seen adjacent to the stent post and originated from outside the sewing ring (Fig. 2). The range of flow convergence seemed to be too large to be caused by TVL alone originating between the stent post and sewing ring. Further examination with CFD revealed that a turbulent flow from the outside of the sewing ring ran through perpendicularly below it and towards the left ventricular outflow tract (LVOT) on the opposite side (Fig. 3, Additional file 2: Video Clip S2), which was different from the turbulent jet shown in Fig. 1. These TEE findings suggested that both PVL and TVL existed in close proximity to each other (Fig. 4). We were convinced that one of the turbulent jets was PVL; therefore, CPB was reinstituted to inspect the prosthesis directly.

**Additional file 1: Video Clip S1.** Midesophageal aortic valve long-axis image in color compare mode showing transvalvular leakage.



**Additional file 2: Video Clip S2.** Midesophageal aortic valve long-axis image in color compare mode showing flow convergence of paravalvular leakage.


An aortic cross-clamp was placed and antegrade cardioplegia was administered into the aortic root to arrest the heart. We monitored the left ventricle with TEE to avoid additional distension because of AR whilst cardioplegia was administered. Inspection of the prosthesis itself indicated that the leaflets, stent post, and sewing ring appeared to be normal within the annulus; however, there was a region adjacent to the anterior stent post near the right coronary ostium where the tip of the curved Pean forceps entered between the sewing ring and the native annulus. The region was consistent with the TEE findings and may have contributed to the observed leakage. The prosthesis was removed by cutting the surgical sutures without dropping the pledgets to the left ventricle, and it was confirmed to be uninjured. Simultaneously, the annulus and pledgets sutured to the annulus were checked. AVR was performed with the same prosthesis again in the supra-annular position using the non-everting mattress suture technique with pledgets, similar to the first AVR. It was verified that there was no loose region between the annulus and the sewing ring before the ascending aorta was closed. After weaning from CPB, TEE was performed immediately, which revealed that the unusual perpendicular turbulent flow had disappeared and only a few small TVLs were observed.

Subsequently, the patient’s course was uneventful, and he was discharged from our hospital after approximately 2 weeks. Postoperative follow-up transthoracic echocardiography showed only mild TVL with no evidence of PVL. The patient gave written consent for publication of clinical reports and echo images.

## Discussion and conclusions

Intraoperative TEE can yield accurate information regarding the site of TVL and/or PVL. Generally, after AVR with a stented bovine pericardial valve, TVLs are small central jets, whereas PVLs have a high velocity, are turbulent, originate from outside the sewing ring with flow convergence, and run obliquely toward the left ventricle. However, in a few case reports, both TVL and PVL have been reported as unusual perpendicular jets [1, 6]. In our case, with a stented bovine pericardial valve, the initial TEE examination revealed a perpendicular turbulent jet diagnosed as TVL originating from between the stent post and the sewing ring (Fig. 1). However, a relatively wide area of flow convergence was observed near the anterior stent post in the ME AV SAX view (Fig. 2). For semi-quantitative evaluation of PVL, the circumferential extent of the jet in the SAX AV view is expressed as a percentage of the total sewing ring circumference (mild, < 10%; moderate, 10–29%; severe, ≧30%) [7]. In the current case, moderate leakage originating from outside the sewing ring was observed, and the circumferential extent of the jet was over 10%. Because TVLs originating from between a stent post and sewing ring show flow convergence from outside of the prosthetic annulus that is usually trivial or mild [1], our case was inconsistent with the range of flow convergence caused by only TVL. Further TEE identified another turbulent flow from the outside of the sewing ring that ran perpendicularly below the sewing ring and biological valves toward the LVOT on the opposite side (Fig. 3). In the current case, both PVL and TVL were seen, with a perpendicular jet direction, originating in close proximity from the site around the anterior stent post near the ostium of the right coronary (Fig. 4). Therefore, it was difficult to distinguish between them.

Typically, TVL originates from the fabric-covered regions of the stent posts or from the region between the stent post and the sewing ring in stented prosthetic valves, because the leakage might be due to a structural problem related to the material of the fabric. This type of leakage spontaneously decreases or even disappears by the end of the surgery after administration of protamine [1]. In the current case, when TEE was performed after weaning from extra CPB, TVL was not observed even without administration of protamine, although AVR was performed using the same prosthetic valve. The fabric region of the prosthetic valve was covered with cellular elements; we speculated that this prevented the leak, as it was used in AVR once and was soaked in blood.

**Fig. 5:**
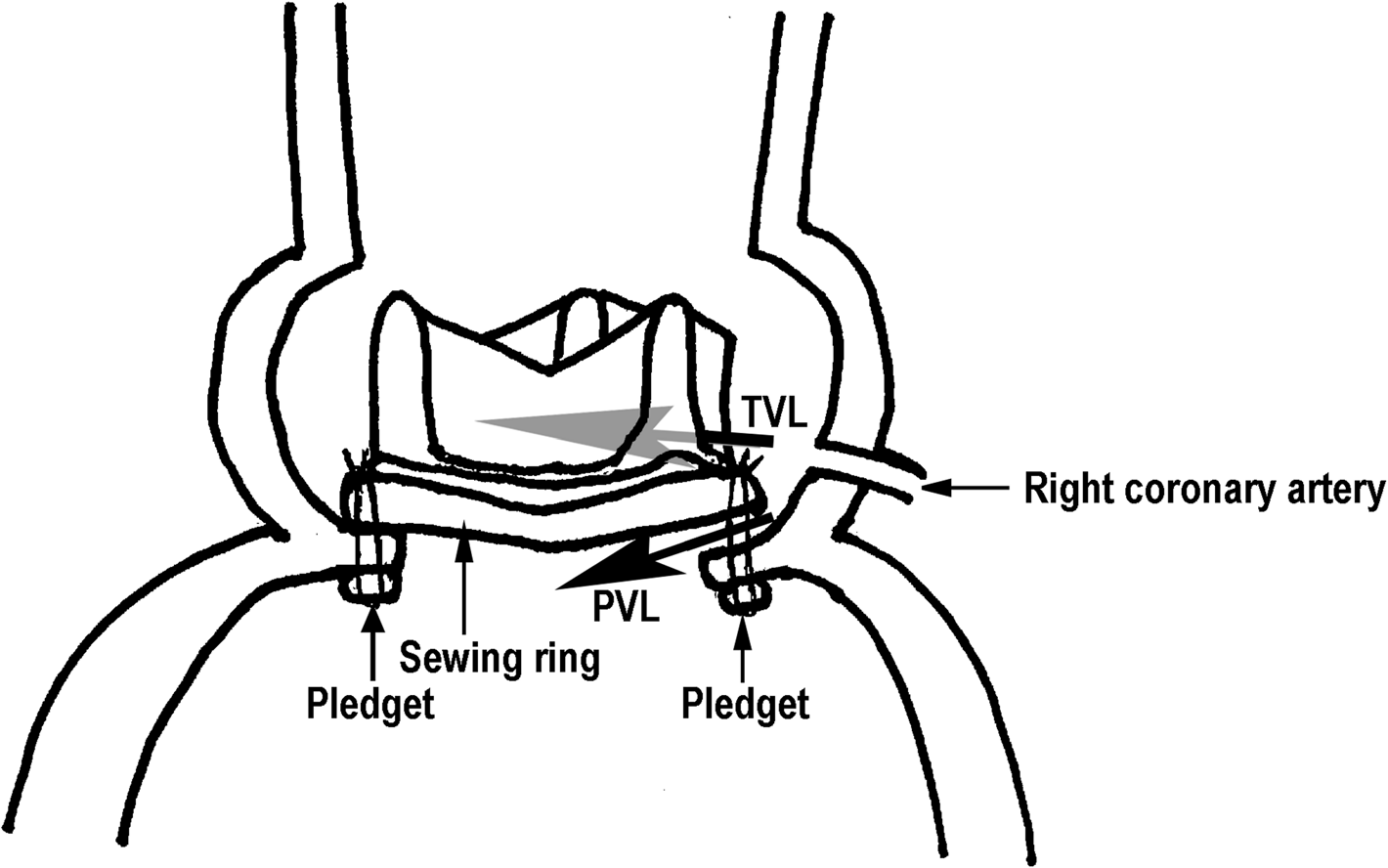
Schematic illustration showing the site of the TVL and the PVL. TVL: transvalvular leakage, PVL: paravalvular leakage

With regard to the surgical procedure, the non-everting mattress technique with pledgets is most frequently used for supra-annular aortic prosthesis, as in the current case. In our case, the space between sewing ring and annular retained native potion resulted in the perpendicular turbulent jet. This surgical procedure may result in perpendicular PVL if a space is formed between the sewing ring and the annular retained potion. Figure 5 shows the sites of the perpendicular turbulent jets of both of PVL and TVL schematically.

In conclusion, perpendicular turbulent flow raises the possibility of both TVL and PVL in the case of AVR with stented bovine pericardial valves. For a differential diagnosis of TVL or PVL, it is important to know the surgical procedures and the morphology of the valve.

## Data Availability

Not applicable.

## Abbreviations

AR: Aortic regurgitation
AV: Aortic valve
AVR: Aortic valve replacement
CFD: Color flow Doppler
CPB: Cardiopulmonary bypass
LAX: Long-axis
LVOT: Left ventricular outflow tract
ME: Midesophageal
PFO: Patent foramen ovale
PVL: Paravalvular leakage
SAX: Short-axis
TEE: Transesophageal echocardiography
TVL: Transvalvular leakage
